# The Effect of Sericin from Various Extraction Methods on Cell Viability and Collagen Production

**DOI:** 10.3390/ijms11052200

**Published:** 2010-05-20

**Authors:** Pornanong Aramwit, Sorada Kanokpanont, Titpawan Nakpheng, Teerapol Srichana

**Affiliations:** 1 Department of Pharmacy Practice, Faculty of Pharmaceutical Sciences, Chulalongkorn University, Bangkok 10330, Thailand; 2 Department of Chemical Engineering, Faculty of Engineering, Chulalongkorn University, Bangkok 10330, Thailand; E-Mail: sorada.k@chula.ac.th; 3 Department of Pharmaceutical Technology and Drug Delivery System Excellence Center, Faculty of Pharmaceutical Sciences, Prince of Songkla University, Hat Yai, Songkla 90110, Thailand; E-Mails: titpawan@gmail.com (T.N.); teerapol.s@psu.ac.th (T.S.)

**Keywords:** sericin, cell viability, collagen, extraction, concentration

## Abstract

Silk sericin (SS) can accelerate cell proliferation and attachment; however, SS can be extracted by various methods, which result in SS exhibiting different physical and biological properties. We found that SS produced from various extraction methods has different molecular weights, zeta potential, particle size and amino acid content. The MTT assay indicated that SS from all extraction methods had no toxicity to mouse fibroblast cells at concentrations up to 40 μg/mL after 24 h incubation, but SS obtained from some extraction methods can be toxic at higher concentrations. Heat-degraded SS was the least toxic to cells and activated the highest collagen production, while urea-extracted SS showed the lowest cell viability and collagen production. SS from urea extraction was severely harmful to cells at concentrations higher than 100 μg/mL. SS from all extraction methods could still promote collagen production in a concentration-dependent manner, even at high concentrations that are toxic to cells.

## 1. Introduction

Extracellular matrix proteins such as collagen, fibronectin, and gelatin are known to play important roles in the attachment and growth of mammalian cells. We recently showed that silk sericin (SS), a high molecular weight granular protein with adhesive and gelatin-like characteristics, can promote growth of the mouse fibroblast cell line L929, as well as activation of collagen production both *in vitro* and *in vivo* [1,2]. Many studies have also demonstrated that SS can accelerate the proliferation and attachment of several mammalian cell lines [3–5], and insect cell culture was also reported to be improved by SS [6]. Moreover, SS added to freezing media as an alternative to fetal bovine serum improved the survival of various cell lines during cryopreservation [7]. However, Terada *et al*. reported that culture supplemented with 1.0% SS resulted in no viable cells, which indicates that the presence of 1.0% SS is harmful to cells [3]. These data demonstrate that the concentration of SS supplemented to culture medium is also a significant factor for cell viability. Nevertheless, the optimal concentration of SS for promoting cell viability has never been reported.

SS can be extracted by various methods, such as high pressure and high temperature techniques, acid or alkaline solutions, or enzyme extraction. The method of extraction significantly affects the biochemical activities of silk proteins. Kurioka *et al*. reported that acid-degraded, alkali-degraded, and hot water-degraded SS powders exhibit different trypsin inhibitory activities and have different isoelectric points [8]. Furthermore, different SS extraction methods alter its amino acid composition, which may influence its cell-growth and collagen secretion in cells.

The objective of this study was to investigate the chemical properties of SS extracted from Thai silk strains via various extraction methods, which have never yet been investigated. In addition, we determined the effect of various concentrations of SS obtained from the different extraction methods on fibroblast cell viability and collagen production. These data yield important fundamental information for further development of SS as a serum-free medium supplement.

## 2. Results and Discussion

It has long been known that SS can accelerate the proliferation of several cell lines, including hybridoma cells [3,5,6,9]. Tsubouchi *et al*. also reported that SS can enhance the attachment of cultured human skin fibroblasts [4]. The attachment and subsequent proliferation of fibroblast cells are considered to play important roles in the healing process of skin lesions. In this study, the L929 mouse fibroblast cell line has been used as a model to investigate the roles of SS from various extraction methods on cell viability and collagen production.

Recently, SS has been shown to have a protective effect against several toxicities, such as alcohol-induced gastric injury, in animal models [10]. However, the SS used in most previous reports [3,5,6,9,10] was extracted by heat or alkaline solution, even though SS can be extracted by various methods, which affect its physical and biological properties, as shown here. This is the first study to compare the enhancement of cell viability by SS at different concentrations derived by extraction procedures. We found that SS does not have only positive effects on cell viability, as at certain concentrations it starts to induce toxicity. This may be an explanation for the previously reported detrimental effects of SS in clinical uses [11,12]. Moreover, extraction methods also play an important role in SS activity. Many studies have used SS prepared by heat or alkaline extraction and have reported the advantageous effects of this protein on cells. This is in agreement with our 3-(4,5-dimethylthiazol-2-yl)-2,5-diphenyltetrazolium bromide (MTT) result, which showed that SS extracted by heat, acid and alkaline solution is rarely toxic to cells at concentrations up to 100 μg/mL. Nevertheless, the extraction method has not previously been emphasized in relation to the use of SS, which may lead to confusion, as we have proven that SS derived by urea extraction method was severely toxic to cells. Since SS has been widely investigated for its use in biomedical applications, this point should be clarified in order to avoid misleading interpretation of results.

### 2.1. Molecular Weight of SS

**Figure 1.**
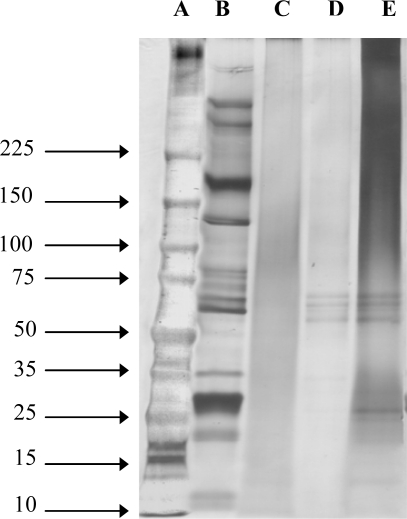
Molecular weight of SS extracted by various methods. (**A**) Marker (**B**) Urea-extracted sericin (**C**) Heat-degraded sericin (**D**) Acid-degraded sericin (**E**) Alkali-degraded sericin.

SS extracted by various methods has different molecular weights, as shown in Figure 1. Different extraction methods provided different molecular weight SS, which may result in different chemical and biological properties. SS extracted with urea showed clear bands with molecular weights ranging from 10 to >225 kDa. Sodium dodecyl sulfate polyacrylamide gel electrophoresis (SDS-PAGE) of acid-degraded and alkali-degraded SS showed distinguishable bands within the range of 50–150 kDa and 15–75 kDa, respectively. However, the number of bands from acid-degraded and alkali-degraded SS were much lower than that of SS extracted with urea. SDS-PAGE of SS prepared by the high temperature and high pressure degumming technique showed broad bands, with molecular weights ranging from 25–150 kDa. Our findings are in agreement with those reported by Sprague, which indicates that SS is a mixture of at least 15 different polypeptide chains, ranging in size from 20 to 220 kDa [13].

According to these studies, SS from heat, acid, alkaline and urea extraction methods show different molecular weights. This may seem insignificant, but it may in fact reflect biological properties of SS, such as its antimicrobial activity. Other researchers have reported that the antimicrobial property of SS is derived from a low molecular weight protein, seroin, from *B. mori* [14,15]. Seroin is not involved in silk fiber construction and may play a role in protecting silk against microbial degradation [15]. Zurovec *et al*. reported that seroin polypeptides are present in silk as 22.5 and 23 kDa molecules, and that these polypeptides are liberated from other proteins when silk components are dissolved [14]. The implication of these findings is that urea extraction should be the only method that would provide SS with antimicrobial activity.

### 2.2. Particle Size and Zeta Potential Measurement

Table 1 shows the zeta potential and particle size of SS from different extraction methods. SS from all extraction methods exhibited negative zeta potential values. Zeta potential of SS from urea extraction yielded the highest negative charge, followed by acid-degraded SS, heat-degraded SS and finally alkali-degraded SS. Alkali-degraded SS had the largest particle size, followed by heat-degraded and acid-degraded SS, while SS extracted by urea solution had the smallest particle size. Since urea-extracted SS is present as a very small-sized compound in water, it may be in soluble form, while SS extracted by other methods may be present as hydrocolloids.

Zeta potential is the potential difference between the dispersion medium and the stationary layer of fluid attached to the dispersed particle. The magnitude of the zeta potential gives an indication of the potential stability of the system, where high zeta potentials (either negative or positive) indicates electrically stabilized particles, while colloids with low zeta potentials tend to coagulate or flocculate [16]. From our results, SS obtained from the alkaline extraction method had more of a tendency to coagulate in this solution, which corresponds to the largest particle size. However, SS obtained by heat, acid and urea extraction were stably dispersed with a lower degree of coagulation compared to alkali-degraded SS. Moreover, SS obtained from urea extraction was the most electrically stable and had the least tendency to coagulate, which was confirmed by the smallest mean particle size.

### 2.3. Amino Acid Analysis

Amino acid content in SS extracted from various methods is shown in Table 2. There were slight variations in the amino acid percentage in SS extracted by different methods; however, the main amino acid component in SS was still the same. Serine was the dominant amino acid in SS (∼30%), while aspartic acid and glycine composed about 10–20%. The amount of methionine found in heat-degraded SS was significantly higher than in SS extracted by other methods, while the amount of tyrosine found in urea-extracted SS was significantly lower than in SS extracted by other methods. Moreover, heat-extracted SS, which contains the highest amount of methionine and cysteine, sulfur-containing amino acids that can generate double-helical structures, can induce the highest levels of collagen production. This result corresponded with our previous report, which showed that methionine in SS relates to its collagen production activity [1].

### 2.4. Cytotoxicity of SS Solution

**Figure 2.**
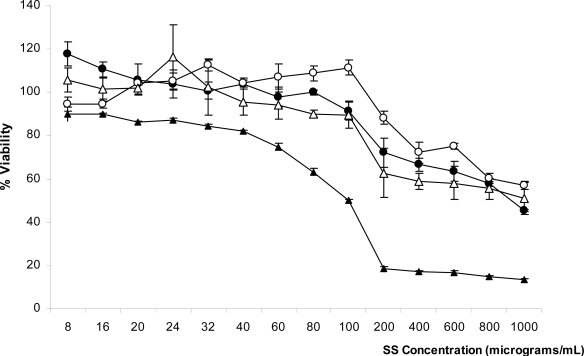
Viability study of L929 cells incubated with SS solutions via the MTT assay after incubation for 24 h. Error bars represent the standard error of the mean (n = 3). (▵) Acid-degraded sericin, (**○**) Alkali-degraded sericin, (**•**) Heat-degraded sericin, (▴) Urea-extracted sericin.

The MTT assay indicated that SS solutions from all extraction methods had no toxicity to mouse fibroblast cells at concentrations up to 40 μg/mL after 24 h incubation (Figure 2). It also indicated that heat-degraded and alkali-degraded SS exhibit the least cell toxicity. SS derived from all extraction methods except urea extraction could promote cell viability at low concentrations. SS from urea extraction showed slight toxicity at concentration as low as 60 μg/mL and its toxicity became significant at concentrations higher than 100 μg/mL, while SS from other extraction methods showed toxicity to a lesser extent than urea-extracted SS, as shown by the percent viability of fibroblasts. These data indicate that extraction method and SS concentration have significant effects on growth and viability of fibroblast cells.

Heat-degraded SS showed the least toxicity to L929 cells at concentrations up to 100 μg/mL, while acid-degraded and alkali-degraded SS showed similar results, but at lower levels of activation, which is consistent with results of other reports [3,5,9]. However, at concentrations higher than 100 μg/mL, viability of L929 cells decreased. Similarly, Terada *et al*. reported that SS from alkaline extraction at low concentrations increased the population in HeLa (human epithelial cell) cultures, while higher concentration of SS (0.3%) did not [3]. This study also reported that SS at 1.0% was severely harmful to the murine hybridoma (2E3-O) cell line [3]. From these data, we can conclude that concentration and the extraction method of SS, as well as the particular cell line, can affect the cell viability.

According to our results, the optimum concentration of SS for promoting cell viability depends on the SS extraction method. SS obtained by urea extraction should not be used as a supplement for serum-free medium, since it is toxic to cells. Heat-degraded and alkali-degraded SS are the most suitable for cell culture with the optimum concentration at 100 μg/mL. Low concentrations of acid-degraded SS were beneficial to cells. Concentrations as low as 8 μg/mL lead to the greatest cell viability.

### 2.5. Adherence of L929 Mouse Fibroblast Cell Line to SS Films

**Figure 3.**
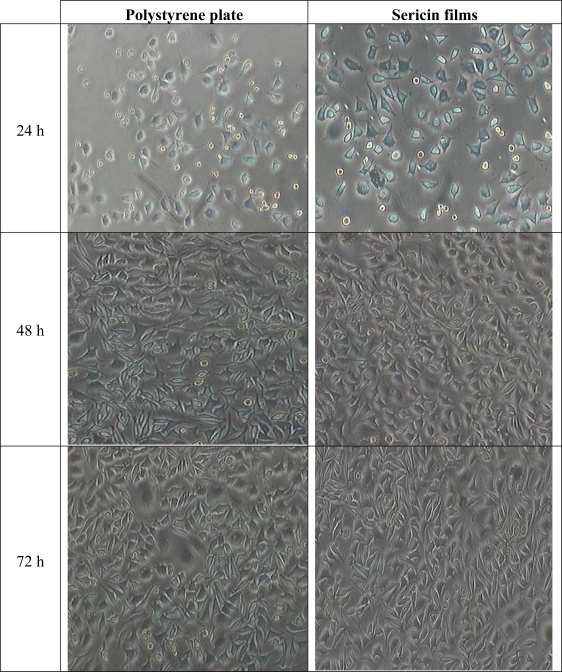
Morphology of L929 mouse fibroblasts cultured on polystyrene culture plates and on SS films at 20X at 24 h, 48 h and 72 h after cell seeding at 20,000 cells/1,257 mm^2^.

Since heat-degraded SS significantly promoted cell growth compared to other extraction techniques, and because of its chemical-free property, heat-degraded SS was used to study the adherence of fibroblast cells on SS films. The morphology of L929 mouse fibroblasts cultured on SS heat-extracted films observed at 24, 48, and 72 h are shown in Figure 3. Cells started attaching to SS films and began proliferating after 24 h similar to cells on styrene culture plates, which were used as a positive control. Approximately 70% of cells on both control and SS plate attached to the surface at 48 h and became confluent after 72 h. After 72 h, cells fully proliferated and formed complete pseudopodia like structures on styrene culture as well as SS plate. Moreover, the number of cells attached on SS plate at 72 h was slightly higher than number of cells on styrene culture plate.

### 2.6. Determination of Soluble Collagen Production Induced by SS

**Figure 4.**
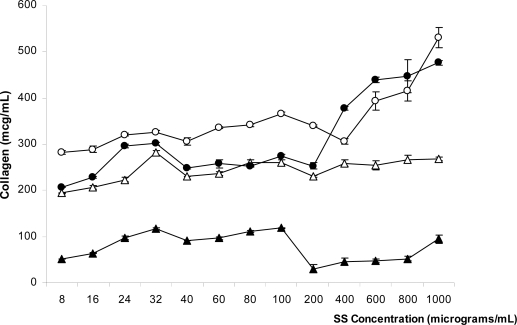
Collagen type 1 production in fibroblast cell line L929 when various SS concentrations were added into the culture medium for 24 h to make the final concentration of SS in each well 8–1,000 μg/mL, respectively. Error bars represent the standard error of the mean (n = 3). (▵) Acid-degrade sericin, (**○**) Alkali-degraded sericin, (**•**) Heat-degraded sericin, (▴) Urea-extracted sericin.

SS extracted by all methods can induce collagen type 1 production from the fibroblast cell line L929 (Figure 4), while the negative control (fibroblast cells without SS as supplement in culture medium) did not produce any collagen (data not shown). However, urea-extracted SS induced the lowest amount of collagen production, compared to SS extracted by the other methods at all concentrations. Heat-degraded SS induced the highest collagen type 1 production at concentrations from 8–200 μg/mL. At concentrations higher than 200 μg/mL, alkali-degraded SS could activate the highest collagen production. However, alkali-degraded and heat-degraded SS at concentrations higher than 200 μg/mL induced significant levels of collagen type 1, but resulted in fewer viable cells. These data indicate that SS at concentrations higher than 200 μg/mL can induce collagen production, even though it is toxic to cells.

Enhancement of fibroblast collagen production in cells is normally related to transforming growth factor (TGF)-β [17], which is generally released only from surviving fibroblast cells 2 h after cells are activated by chemicals or trauma, and reaching peak levels after 12 h [18]. This supports our result that collagen content in cell culture still increased at SS concentrations higher than 100 μg/mL, even though the percentage of cell viability decreased. Collagen production may be generated from fibroblast cells, which are activated by silk protein at an early stage when most cells are still viable before SS becomes toxic to cells.

## 3. Experimental Section

### 3.1. Materials

#### 3.1.1. Silkworm Cocoons

Fresh *Bombyx mori* cocoons were kindly supplied by Chul Thai Silk Co., Ltd. (Petchaboon province, Thailand). Native Thai silkworms, white cocoons, were produced in a controlled environment.

#### 3.1.2. Fibroblast Cell Culture

The mouse fibroblast cell line L929 (Chinese Academy of Preventive Medical Sciences, Beijing, China) was cultured in Dulbecco Modified Eagle Medium (DMEM) containing 10% fetal bovine serum (FBS) and antibiotics (100 U penicillin and 100 U streptomycin per mL) under 5% CO_2_ at 37 °C. The medium was changed every 2 days. When cells reached confluence, they were harvested using 0.25% trypsin-EDTA (Gibco^®^, California, USA), followed by addition of fresh culture medium to create a new single cell suspension for further incubation.

#### 3.1.3. Preparation of SS Powder Using a High Temperature and High Pressure Degumming Technique (Heat-Degraded SS Powder)

Cocoons of *B. mori* silkworms were cut into square pieces and extracted with purified water by autoclaving (SS-320, Tomy Seiko Co., Ltd., Tokyo, Japan) at 120 °C for 60 min. The aqueous solution obtained from autoclaving silk cocoons was collected and called heat-degraded SS. The aqueous solution was then filtered to remove insoluble material, which is fibroin. After that, the filtrate was frozen and lyophilized using a Heto LL 3000 lyophilizer (Allerod, Denmark) to obtain SS powder. The SS molecular weight from all strains was estimated by SDS-PAGE.

#### 3.1.4. Preparation of SS by Citric Acid and Sodium Carbonate Solution (Acid-Degraded and Alkali-Degraded SS Powders)

Acid-degraded and alkali-degraded SS powders were extracted using a previously described method by Kurioka *et al*. with some modifications [8]. For acid-degraded SS powder preparations, cocoons were cut and added to a 1.25% citric acid solution, then boiled for 30 min. After removing insoluble fibers by paper filtration, the clear filtrate was immediately dialyzed in distilled water for three days using cellulose tubing (Cellusep T2, MWCO 6,000–8,000, Sequin, Texas, USA) and distilled water was changed regularly. The pH of the final solution was measured to verify complete removal of citric acid. The SS solution was then frozen and lyophilized. Alkali-degraded SS powder was prepared similarly, using 0.5% sodium carbonate solution instead of citric acid.

#### 3.1.5. Preparation of SS by Urea Solution

SS extracted by urea solution was prepared using a previously described method with some modifications [4]. Freshly cut cocoon shells were soaked into 8 M urea aqueous solution for 30 min and then refluxed at 85 °C for 30 min. Centrifugation and filtration were performed to remove all insoluble residues. The solution was thoroughly dialyzed in distilled water using cellulose tubing (Cellusep T2, MWCO 6,000–8,000, Sequin, Texas, USA) for three days and distilled water was changed regularly. The pH of final solution was measured to verify complete removal of urea solution. The SS solution was frozen and then lyophilized.

### 3.2. Methods

#### 3.2.1. Molecular Weight Determination

To determine the molecular weight of SS, polyacrylamide gel electrophoresis was performed as previously described with some modifications [19]. Briefly, sample solutions for SDS-PAGE were prepared by adding an equal volume of sample buffer (0.25 M Tris-HCl, pH 7.0 containing 4% SDS, 10% sucrose, 10% 2-mercaptoethanol, and 0.025% bromophenol blue) to each protein solution. Each sample solution was then incubated at 98 °C for 2–3 min and loaded onto a 5%–20% gradient gel (Atto Corporation, Tokyo, Japan). Electrophoresis was performed in 125 mM Tris base with 0.96 M glycine and 0.5% SDS, polypeptide bands were detected by silver staining.

#### 3.2.2. Particle Size and Zeta Potential Measurement

The size of self-aggregates was measured by a dynamic light scattering method based on the particle size option in a Zetasizer Nano-ZS (ZEN 3600, Malvern Instruments Ltd., Worcestershire, UK). The scattered intensity was registered at a scattering angle of 90° at 25 °C. Zeta potentials were measured by a Zetasizer Nano-ZS instrument with palladium-coated electrodes. All samples were adjusted to pH 7.0 prior to particle size and zeta potential measurement. The zeta potential presented is the average value of analyses in triplicate.

#### 3.2.3. Amino Acid Analysis

SS amino acid compositions were measured with an amino acid analyzer (Hitachi L-8500A, Tokyo, Japan). Samples were hydrolyzed in 4 M methanesulfonic acid containing 0.2% 3-(2-aminoethyl) indole (Wako Pure Chemical Industries, Ltd., Tokyo, Japan) at 100 °C for 24 h under vacuum prior to amino acid analysis. All experiments were performed in triplicate.

#### 3.2.4. Cytotoxicity of SS Solution

L929 mouse fibroblast cells at an initial concentration of 2 × 10^4^ cells/well were seeded in a 96-well plate in DMEM containing 10% FBS. After 24 h, the culture medium was replaced with fresh medium. SS solutions of various concentrations in purified water were filter sterilized by 0.22 μm membrane filter (Sartorius Ltd., Epsom, UK) prior to adding to the culture medium to give final SS concentrations in each well at 8.0–1000 μg/mL. Cells without SS solution served as negative controls. Melittin, a peptide from bee venom toxin, from 0.125 to 1.0 mg/mL, was used as a positive control. After incubation for 24 h, MTT assay was performed to evaluate cell activity [20]. The absorbance was determined by a microplate reader (Biohit 830, Biohit^®^, Helsinki, Finland) at a wavelength of 570 nm. The percentage of viable cells was calculated and compared to the negative control. All experiments were done in triplicate.

#### 3.2.5. Adherence of L929 Mouse Fibroblast Cell Line on SS Films

Heat-degraded SS films were cast from SS solution (0.1% w/v, in water pH 5) in polystyrene 12-wells cell culture plates (well diameter 20 mm). After air-drying, the films were crosslinked by ultraviolet (UV) irradiation for 60 min and sterilized with 70% ethanol followed by phosphate-buffered saline (PBS, pH 7.5) before seeding cells. L929 mouse fibroblast cells were seeded onto the sterilized films (2 × 10^4^ cells/well). Cells were cultured in DMEM containing 10% FBS and antibiotics (100 U penicillin and 100 U streptomycin per mL) under 5% CO_2_ at 37 °C. Cells were harvested at 24, 48 and 72 h, and the morphology of cells on culture plates (control) and on films coated on culture plates were observed by light microscopy (Nikon, TS100, Melville, New York, USA). All experiments were done in triplicate.

#### 3.2.6. Determination of Soluble Collagen Production Induced by SS

L929 mouse fibroblast cells were cultured at the same cell content and method as for the cytotoxicity study of SS solution. Cells without SS solution served as a negative control. Supernatants were collected after cell incubation for 24 h. The total amount of soluble collagen type 1 was assayed using the Sircol^®^ collagen assay kit (Biocolor Ltd., Northern Ireland, UK). The results were determined by a microplate reader (Biohit 830, Biohit^®^, Helsinki, Finland) at a wavelength of 500 nm. All experiments were done in triplicate. The amount of collagen was calculated based on a standard curve of soluble collagen (standard bovine collagen type 1, produced from USA disease free animals).

## 4. Conclusions

SS can promote cell viability at certain concentrations, but it can be toxic to cells at higher concentrations. The method of extraction of SS also has significant effects on cell viability. Urea-extracted SS showed the lowest cell viability compared to SS extracted by heat, acid and alkaline methods. Urea-extracted SS was severely harmful to cells at concentrations higher than 100 μg/mL. Heat-degraded SS activated the highest collagen production, while urea-extracted SS showed the lowest level of collagen activation. SS from all extraction methods could still promote collagen production in a concentration-dependent manner, even at high concentrations that are toxic to cells, which indicate that collagen was generated before fibroblast cells departed.

## Figures and Tables

**Table 1.** Zeta potential and particle size of SS from different extraction methods.

**Extraction method**	**Zeta potential (mV)**	**Mean size (nm)**
Heat	−20.69 ± 2.14	110.42 ± 35.07
Acid	−32.12 ± 5.26	23.80 ± 16.07
Alkaline	−15.87 ± 2.89	824.42 ± 86.67
Urea	−68.36 ± 5.67	4.62 ± 2.44

**Table 2.** Amino acid composition of SS extracted using various methods (in mole%).

**Amino acid**	**Extraction method of SS**			
	**Heat**	**Urea**	**Acid**	**Alkaline**
Asp	15.64	18.31	15.93	19.88
Ser	33.63	31.27	31.86	30.01
Glu	4.61	5.27	5.75	5.93
Gly	15.03	11.23	10.49	11.01
His	1.06	3.26	2.47	1.72
Arg	2.87	5.41	4.92	4.92
Thr	8.16	8.36	8.51	6.49
Ala	4.10	4.33	3.72	4.21
Pro	0.54	1.46	0.78	1.24
Cys	0.54	0.39	0.53	0.23
Tyr	3.45	0.36	5.56	5.24
Val	2.88	2.96	2.95	2.94
Met	3.39	0.12	0.06	0.15
Lys	2.35	3.14	3.48	2.89
Ile	0.56	0.96	0.87	0.75
Leu	1.00	1.58	1.43	1.56
Phe	0.28	0.60	0.71	0.81
